# Humbugging in Medicine

**Published:** 1897-08

**Authors:** 


### Humbugging in Medicine.

It is a very sad commentary on the practice of medicine that humbugging is almost a necessity, and the honest physician, that is, the physician who takes the patient into his confidence, soon finds himself without that patient. As a man once said to his doctor: "Doctor, my wife thinks she has some trouble with her lung, and if you do not humor her some one else will." The trouble is that if a physician says honestly to a patient that she is well and needs no medical attention, she straightway writes him down for a fool and sends for some one else. This one may be just as honest as the poor fellow who was dismissed, but he holds his tongue where the other one talked and "looks wise, feels foolish and says nothing." Patients always like to think their physician is above them in knowledge, and when that familiarity which is said to breed contempt is once established between doctor and patient, obedience and respect are lost. The patient need not understand all that is done, nor need the nature or proposed effect of the medicine given be revealed. Indeed the truly honest man can do his patient justice and himself credit by explaining nothing and using his best effort to effect a cure or an improvement. The public knows too much of medicine as it is, and this smattering should not be encouraged by any confidence of the physician. One can be honest and also discreet. —*Maryland Medical Journal.*

